# Genome-Wide Identification of Cotton (*Gossypium* spp.) Trehalose-6-Phosphate Phosphatase (TPP) Gene Family Members and the Role of *GhTPP22* in the Response to Drought Stress

**DOI:** 10.3390/plants11081079

**Published:** 2022-04-15

**Authors:** Weipeng Wang, Hua Cui, Xiangfen Xiao, Bingjie Wu, Jialiang Sun, Yaxin Zhang, Qiuyue Yang, Yuping Zhao, Guoxiang Liu, Tengfei Qin

**Affiliations:** 1 Key Laboratory of Tobacco Improvement and Biotechnology, Tobacco Research Institute of Chinese Academy of Agricultural Sciences, Qingdao 266100, China; wweipeng125@gmail.com; 2 College of Agriculture, Liaocheng University, Liaocheng 252059, China; wbj8258033@163.com (B.W.); sjl5845@126.com (J.S.); 18339511268@163.com (Y.Z.); yqiuyue@163.com (Q.Y.); zzyp000@126.com (Y.Z.); 3 Key Laboratory of Cell and Gene Circuit Design, Shenzhen Institute of Synthetic Biology, Shenzhen Institute of Advanced Technology, Chinese Academy of Sciences, Shenzhen 518055, China; hua.cui@siat.ac.cn; 4 Shanghai Institutes for Biological Sciences, Chinese Academy of Sciences, Shanghai 201602, China; xxf0614@163.com; 5 Beijing Capital Agribusiness Future Biotechnology, Beijing 100088, China

**Keywords:** cotton, trehalose-6-phosphate phosphatase (TPP), gene expression, drought stress, trehalose 6-phosphate (T6P)

## Abstract

Trehalose-6-phosphate phosphatase (TPP) is a key enzyme involved in trehalose synthesis in higher plants. Previous studies have shown that TPP family genes increase yields without affecting plant growth under drought conditions, but their functions in cotton have not been reported. In this study, 17, 12, 26 and 24 TPP family genes were identified in *Gossypium arboreum*, *Gossypium raimondii*, *Gossypium barbadense* and *Gossypium hirsutum*, respectively. The 79 TPP family genes were divided into three subgroups by phylogenetic analysis. Virus-induced gene silencing (VIGS) of *GhTPP22* produced TRV::*GhTPP22* plants that were more sensitive to drought stress than the control plants, and the relative expression of *GhTPP22* was decreased, as shown by qRT–PCR. Moreover, we analysed the gene structure, targeted small RNAs, and gene expression patterns of TPP family members and the physicochemical properties of their encoded proteins. Overall, members of the TPP gene family in cotton were systematically identified, and the function of *GhTPP22* under drought stress conditions was preliminarily verified. These findings provide new information for improving drought resistance for cotton breeding in the future.

## 1. Introduction

Trehalose is a nonreducing disaccharide that is common in bacteria, fungi, yeasts, invertebrates and plants. In addition to being a carbon source and structural component, trehalose is a good protective agent against stress under adverse conditions such as high salinity, hypoxia and nutrient starvation [1,2]. Recent studies have found that many species, such as drought-tolerant cypress, can protect themselves by synthesizing trehalose under adverse conditions such as drought and freezing [3]. An increase in trehalose reserves in plants helps them maintain their metabolism at an extremely low state under abiotic stress conditions and thus plays an indispensable protective role in plants.

The trehalose biosynthetic pathway in plants mainly involves the synthesis of trehalose 6-phosphate (T6P), which is catalysed by trehalose-6-phosphate synthase (TPS), followed by the formation of trehalose, which is catalysed by trehalose-6-phosphate phosphatase (TPP) [4,5]. Hilde et al. found that overexpression of the trehalase (TRE) gene *TRE1* could increase the drought tolerance of *Arabidopsis thaliana* and that *TRE1* is involved in abscisic acid (ABA)-induced stomatal closure [6]. Tobacco plants overexpressing TPS were significantly more tolerant to drought and more resistant to salt, but some of these tobacco plants displayed growth and differentiation defects, such as dwarfism and abnormal root differentiation. Based on the above results, scientists have hypothesized that trehalose and its metabolites are likely involved in the regulation of plant growth and differentiation and also enhance stress tolerance [7,8].

As a key enzyme involved in trehalose synthesis, TPP can improve crop yields in response to abiotic stresses and plays an important role in plant growth and development [9,10,11,12,13,14,15]. For example, in rice, both *OsTPP1* and *OsTPP2* exhibit TPP enzyme activity, and the expression of *OsTPP1* is rapidly upregulated in response to salt and ABA treatment, while the induction effect of cold treatment is relatively slow [16]. As another example, deletion of *RAMOSA3* (*RA3*), a TPP homologue, in maize alters pistil branching and stamen inflorescence differentiation, and the trehalose content in inflorescence primordia in the deletion mutant *ra3* is very low [17]. *Mads* is the promoter that drives the expression of *OsTPP1* in maize, and overexpression of *Mads6* in maize ears can significantly improve maize yields under drought conditions [18]. Taken together, the above-described results show that the expression of TPP gene family members is closely related to cold, drought, ABA levels, etc., and that their expression varies among organs and developmental stages [19].

Cotton is planted worldwide and provides a natural fibre source for the textile industry. The allotetraploid cotton *G. hirsutum* and *G. barbadense* are the main cultivated species that have two sets of subgenomes: At and Dt [20]. If the role of TPP in cotton is similar to that in rice and maize, TPP will clearly bear great value for improving the stress tolerance of cotton. To date, no studies on TPP family members in cotton have been reported. In this study, to deeply investigate evolution, gene expression patterns and the function response to stress of the TPP family genes in cotton growth, development and stress response, TPP family members were comprehensively and systematically identified by using genome sequencing data [21,22,23]. The physicochemical properties, subcellular localization prediction, collinearity analysis, cis-element analysis, and miRNA prediction were performed in this study, and the functions of *GhTPP22* were also preliminary verified in *G. hirsutum*. The results provide a theoretical basis for subsequent studies on the functions of TPP family genes.

## 2. Results

### 2.1. Identification and Fundamental Analysis of TPP Genes

By combining the BLASTP, CD-search and HMMER results, we identified a total of 79 TPP family genes in the four cotton species: 17 in *G. arboreum*, 12 in *G. raimondii*, 24 in *G. hirsutum* and 26 in sea island cotton (*G. barbadense*). To facilitate description, these genes were renamed according to their chromosomal positions. The amino acid lengths of the TPP family proteins ranged from 134 to 422, the average relative molecular weight was 39.54 kDa, and the average isoelectric point was 8.41 (Table 1). Subcellular location prediction indicated that TPP genes were distributed in almost all organelles and most were located in chloroplasts, which illustrated their functional diversity and complexity.

### 2.2. Phylogenetic Analysis of TPP Family Genes

**Figure 1:**
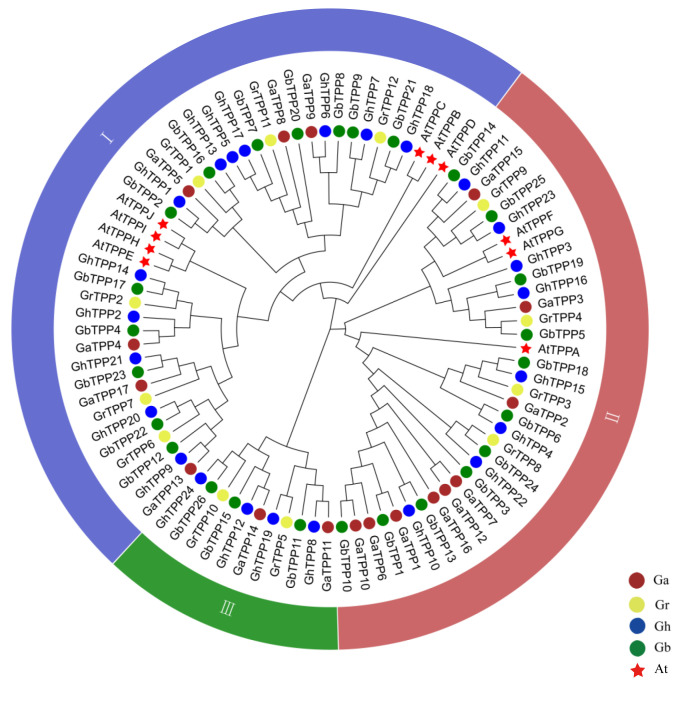
Phylogenetic analysis of TPP family genes in cotton and *Arabidopsis*. The brown, yellow, blue, and green circles represent *G. arboreum*, *G. raimondii*, *G. hirsutum* and *G. barbadense*, respectively, and the red stars represent *Arabidopsis*. Groups I–III are shown in blue, red, and green colour, respectively.

The protein sequences encoded by the TPP gene family members in *Arabidopsis* (10 TPP family genes) and cotton were aligned using MEGA 7.0 to construct a phylogenetic tree. The phylogenetic tree divided the TPP family genes in *Arabidopsis* and cotton into three subgroups, and groups I to III are indicated in blue, red, and green colour, respectively in Figure 1. Genes assigned to the same group were more closely related. The TPP family genes in the At subgroup of *G. hirsutum* and *G. barbadense* were closely grouped on the same branch as the TPP family genes of *G. arboreum*. Similarly, the TPP genes in the Dt subgroup were grouped on the same branch as the TPP family genes of *G. raimondii*. This phenomenon also confirmed the origin of *G. barbadense* and *G. hirsutum* [22]. No TPP genes of *Arabidopsis* were found in group III. According to the evolutionary tree, *G. hirsutum* and *G. barbadense* are more easily divided into the same branch, indicating the closer relationship between these two species.

### 2.3. Conserved Motif and Gene Structure Analyses of TPP Proteins

**Figure 2:**
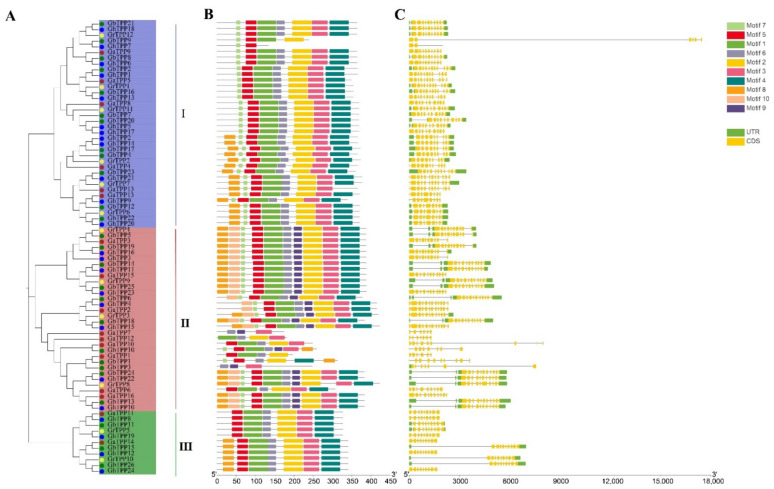
Conserved motifs and structure of TPP genes in cotton. (**A**) The phylogenetic tree was constructed using the protein sequences encoded by 79 TPP genes in cotton. The TPP genes were divided into three groups based on the evolutionary tree. (**B**) The conserved motifs in TPP genes were predicted, and the different colours represent different motifs. (**C**) The intron/exon structures of the TPP genes were analysed. The yellow boxes represent exons, the grey lines represent introns, and the green boxes represent untranslated regions (UTRs).

To understand the functions of TPP family genes, we analysed their conserved sequences and structures in the four cotton species. A total of 79 TPP family genes in the four cotton species could be divided into groups I, II, and III in the phylogenetic tree (Figure 2A), and these groups contained 36, 32, and 11 genes, respectively.

The structures of the TPP gene family were complex (Figure 2C); more than 5 exons were observed in all TPP gene family members except *GhTPP7*, and 53 genes had more than 10 exons. Conserved motif analysis of TPP family genes performed with MEME (Figure 2B) revealed that motifs 1–6 were present in most members, indicating that these motifs are chronologically conserved motifs in the family and may play a role in maintaining the basic functions of the family members. Different TPP genes differ somewhat in terms of their structure and conserved sequences, but TPPs on the same branch have similar conserved sequences, such as *GrTPP4*-*GhTPP23*, which contains all the identified motifs. As shown in Figure 2, differences in gene structure and conserved motifs were found among groups, whereas genes on the same branch were conserved. Information on each motif is provided in Table S1.

### 2.4. Chromosomal Distribution and Homology Analysis of TPP Genes

**Figure 3:**
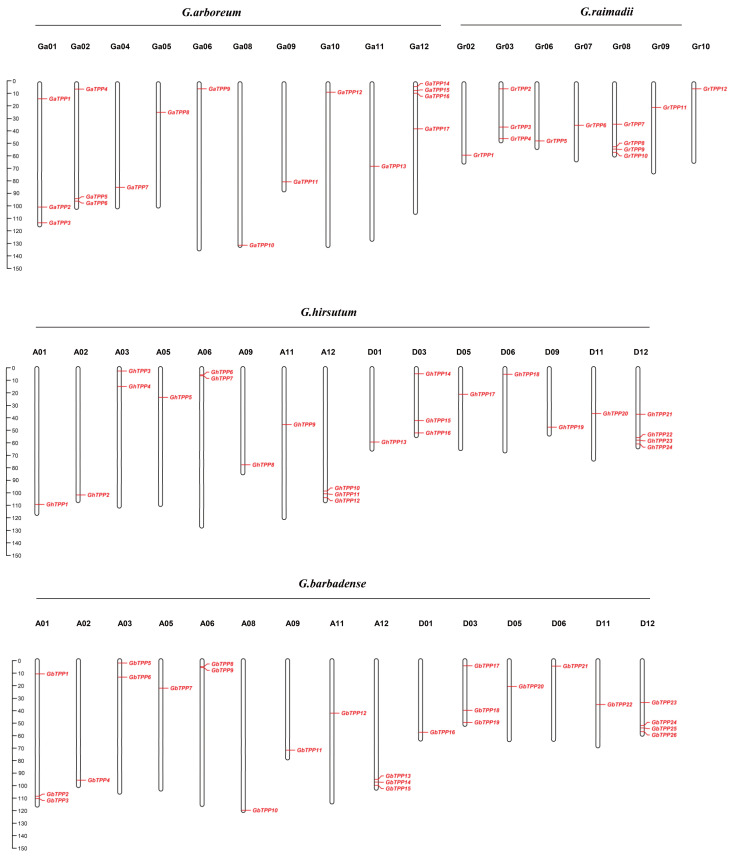
Chromosomal distribution of TPP genes. The red gene ID locations correspond to the positions on the chromosomes; the scale of the chromosomes represents millions of base pairs (Mb).

Based on the structural annotation information of the 79 TPP family genes, we generated chromosomal distribution maps for *G. arboreum*, *G. raimondii*, *G. hirsutum* and *G. barbadense* (Figure 3). In the *G. arboreum* A genome, 17 TPP family genes were located on 10 chromosomes; of these chromosomes, Ga01, Ga02 and Ga12 contained 3, 3 and 4 *GaTPPs*, respectively, and the remaining 7 *GhTPPs* were distributed across the other 7 chromosomes. A total of 12 *GrTPPs* were located on 7 chromosomes in the *G. raimondii* D genome, of which the Gr03 and Gr08 chromosomes contained 3 and 4 *GrTPPs*, respectively. The remaining 5 *GrTPPs* were located on Gr02, Gr06, Gr07, Gr09, and Gr10. In *G. hirsutum*, 24 *GhTPPs* were localized to 15 chromosomes; chromosomes A12, D03, and D12 contained 3, 3, and 4 *GhTPPs*, respectively; and the remaining 12 chromosomes had only 1 or 2 *GhTPPs*. A total of 26 *GbTPPs* were found on 15 chromosomes in *G. barbadense*, with 3, 3, 3 and 4 *GbTPPs* located on chromosomes A01, A12, D03 and D12, and the remaining 14 *GbTPPs* were located on 11 chromosomes. Differences in chromosomes A01, A08 and D09 were found between *G. hirsutum* and *G. barbadense*, which suggested that duplication and deletion possibly occurred during evolution.

**Figure 4:**
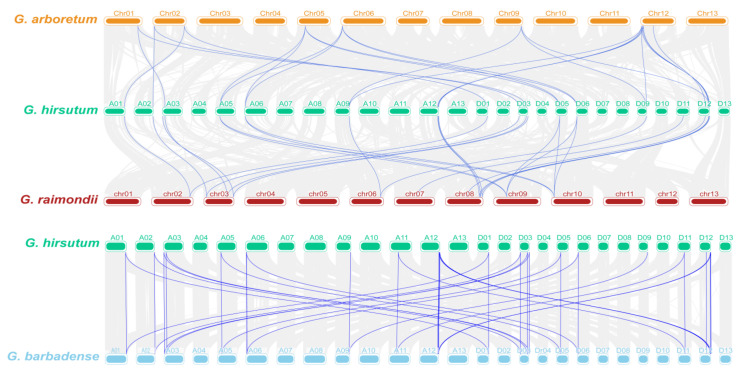
Collinearity analysis of the TPP family members in cotton. From top to bottom, the collinear relationships between *G. arboreum* and *G. hirsutum*, between *G. barbadense* and *G. raimondii*, and between *G. hirsutum* and *G. barbadense* are shown. The grey lines in the background show the collinearity of the entire genome, and the blue lines indicate the collinearity of the TPP genes.

To further explore the evolutionary relationship of the TPP gene family in cotton, we identified all homologous genes between *G. hirsutum* and *G. arboreum*, *G. hirsutum* and *G. raimondii*, and *G. hirsutum* and *G*. *barbadense* and then analysed the collinearity of the TPP family genes (Figure 4). The results showed that no tandem duplications occurred in the four cotton species. The 24 TPP family genes of *G. hirsutum* had corresponding homologues in *G. arboreum* and *G. raimondii*, among these genes, 19 TPP genes had homologues in both *G. arboreum* and *G. raimondii*, 2 had homologues only in *G. arboreum*, and 3 had homologues only in *G. raimondii*. The collinear relationships between *G. barbadense* and *G. hirsutum* indicated that *GbTPP1* and *GbTPP10* had no corresponding homologues in *G. hirsutum*; thus, these genes might have originated from independent events during the evolutionary process of *G. barbadense* and TPP genes may have evolved divergently in *G. barbadense*.

### 2.5. Prediction and Analysis of Cis-Acting Elements in Promoter Regions

**Figure 5:**
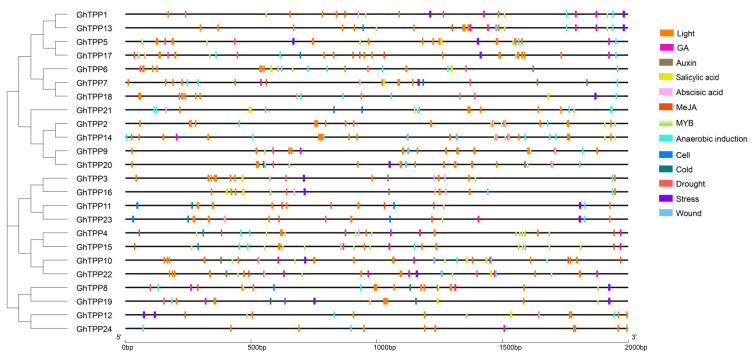
Cis-element analysis of the TPP genes in *G. hirsutum*. The black line represents the 2000-bp sequence upstream of the CDS, and squares of different colours represent various cis-elements. The evolutionary tree is shown on the left.

Cis-acting elements can influence and regulate gene expression by binding to transcription factors. We predicted and analysed the cis-acting elements within the promoter regions of the TPP family genes in *G. hirsutum* and found that TATA boxes, CAAT boxes and other typical cis-acting elements were present in the promoter regions of TPP genes. The relevant details are presented in Table S2. We investigated the cis-acting elements related to hormones and stress responses (Figure 5); based on the analysis, the promoter regions of TPP genes in *G. hirsutum* did not contain elements related to the response to high-temperature stress but contained a large number of light signal response elements (G-boxes, GT1 motifs, TCT motifs, etc.). With the exception of *GhTPP5/8/11/17/19*, all TPP genes contained ABA-associated elements (ABREs). Most members of the TPP family contained various hormone-responsive elements as well as environmental stress-responsive elements, e.g., gibberellic acid (GA) response elements (GARE motifs, P-boxes and TATC boxes), indoleacetic acid (IAA) response elements (AuxRR cores and TGA elements), methyl jasmonate (MeJA) response elements (CGTCA motifs and TGACG motifs), salicylic acid (SA) response elements (TCA elements), drought response elements (MBSs), low-temperature response elements (LTRs), defence elements, stress response elements (DRSs) and antioxidant response elements (AREs). It is hypothesized that the TPP family genes of *G. hirsutum* may be involved in the response to hormone regulation and resistance to abiotic stress.

### 2.6. Prediction of miRNAs Targeting TPP Family Genes

**Figure 6:**
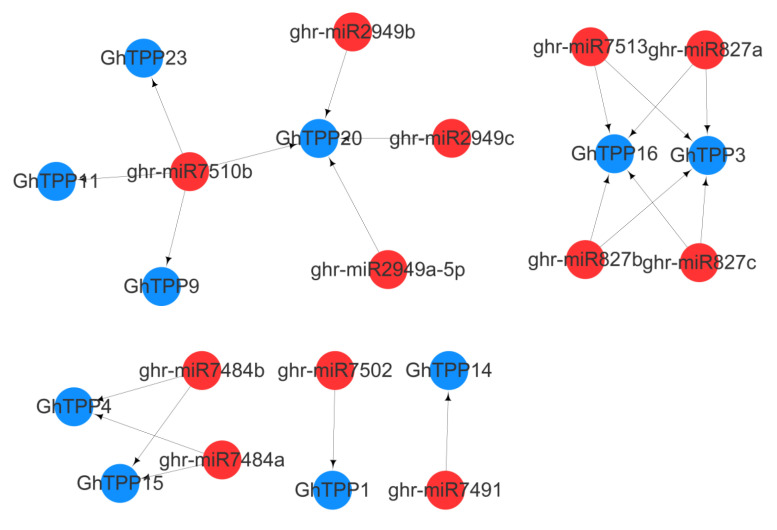
Prediction of miRNAs targeting TPP genes. The red circles reflect the predicted miRNAs, and the blue circles depict the targeted TPP genes. The lines between the circles represent their connections.

The miRNAs are a class of noncoding single-stranded RNA molecules that have a length of approximately 22 nucleotides, are encoded by endogenous genes and are involved in gene expression regulation and responses to biotic and abiotic stress in plants. We predicted miRNA-targeting gene networks of TPP family genes in *G. hirsutum* (Figure 6) and identified a total of 21 interaction relationships. The interaction network comprised 12 miRNAs, and 10 TPP family genes were targeted. Among the genes, *ghr-miR7510b* was involved in regulation of the expression of four TPP genes, *GhTPP9/11/20/23*. *GhTPP3*, *GhTPP16*, and *GhTPP20* are each targeted by four miRNAs, and *GhTPP3* and *GhTPP16* are targeted by the same miRNAs. *GhTPP1, GhTPP4, GhTPP14* and *GhTPP15* are the target genes of *ghr-miR7484b*, *ghr-miR7484a*, *ghr-miR7502* and *ghr-miR7491*, respectively.

### 2.7. Analysis of the Expression Patterns of TPP Family Members in Response to Abiotic Stress

Previous studies have confirmed that plants can respond to abiotic stresses through the synthesis of trehalose. Therefore, studying the expression patterns of genes that encode TPPs under various stresses is very important. Therefore, we obtained and analysed the expression patterns of TPP family members in TM-1 plants under cold, heat, drought and salt stress conditions.

**Figure 7:**
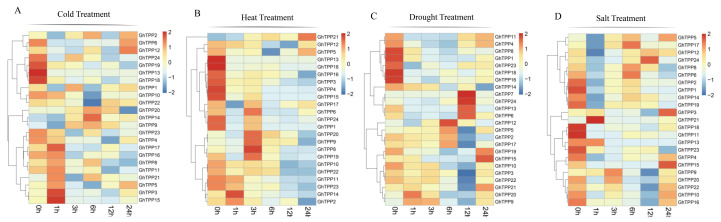
Expression profiles of TPP genes in *G. hirsutum* under different stress treatments. The transcriptomic data were normalized via log2 (FPKM+1) to generate a heatmap. Shown from left to right are the data under cold (**A**), heat (**B**), drought (**C**), and salt (**D**) stress are shown from left to right.

The expression patterns of TPP family genes differed under the same stress conditions, and the expression patterns of the same genes also differed under different stresses. The expressions of *GhTPP23*, *GhTPP4*, *GhTPP17*, *GhTPP16*, *GhTPP8*, *GhTPP11*, *GhTPP21*, *GhTPP5*, *GhTPP3*, and *GhTPP15* were induced after cold treatment for 1 h (Figure 7A) and may thus be more sensitive to cold stress. *GhTPP17*, *GhTPP6*, *GhTPP24*, *GhTPP1*, *GhTPP20*, *GhTPP9*, *GhTPP8*, and *GhTPP19* were upregulated after 3 h of high-temperature treatment, and the expression of *GhTPP21* continued to increase with increasing high-temperature treatment (Figure 7B). The expression levels of *GhTPP11*, *GhTPP4*, *GhTPP8*, *GhTPP1*, *GhTPP23*, *GhTPP16*, and *GhTPP18* were low during the first 6 h under drought stress but began to increase after 12 h. *GhTPP7*, *GhTPP24*, *GhTPP13*, and *GhTPP6* were highly expressed at 12 h, while *GhTPP12*, *GhTPP5*, *GhTPP2*, and *GhTPP17* were upregulated at 6 h. The expression levels of the remaining eight genes (*GhTPP19, GhTPP15 GhTPP10*, *GhTPP3*, *GhTPP22*, *GhTPP21, GhTPP20*, and *GhTPP9*) ranged from high to low, but the expression of these genes increased later (Figure 7C). Compared with other stresses, TPP genes were not sensitive to the onset of salt stress (Figure 7D); most genes were expressed at low levels before 6 h, and *GhTPP4*, *GhTPP15*, *GhTPP9*, *GhTPP20*, *GhTPP22*, *GhTPP10* and *GhTPP16* did not play a role until 24 h. In summary, the results indicated that the expression of most TPP genes was induced in response to different stresses.

### 2.8. Expression Pattern Analysis in Different Tissues and at Different Growth Stages

To further understand the specific functions of TPP family genes in cotton growth and development, we downloaded and analysed the transcriptomic data of TPP family genes for ovule tissue at −3/0/1/3/5/10/15/20/25 days post-anthesis (DPA); fibre tissue at 10/15/20/25 DPA; and anther, bract, filament, leaf, petal, pistil, root, sepal, stem and torus tissues from TM-1 plants.

**Figure 8:**
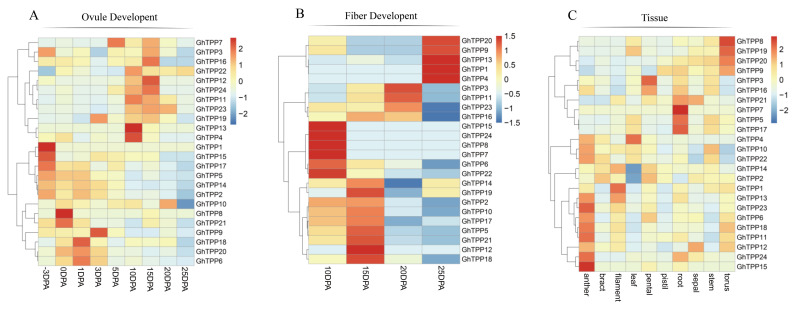
TPP gene expression profiles during different developmental periods and in different tissues of *G. hirsutum*. The transcriptomic data were normalized via log2 (FPKM+1) to generate a heatmap. (**A**) Expression of TPP genes in *G. hirsutum* at different stages of ovule development. (**B**) Expression of TPP genes at different stages of *G. hirsutum* fibre development. (**C**) Expression of TPP genes in different tissues.

During ovule development, the TPP family genes could be divided into two groups based on a cluster analysis (Figure 8A). The first group of TPP genes (*GhTPP7-GhTPP4*) were upregulated at 10 DPA and 15 DPA, whereas the other group of TPP family genes were functional at the fibre initiation stage (−3 to 3 DPA). During fibre development, TPP family genes were expressed at all four time points (10/15/20/25 DPA), and each gene showed high expression at only one time point (Figure 8B). In conclusion, TPP family genes are expressed at both the initiation and elongation stages of cotton fibres, and different TPP genes function at different time points.

The expression levels of different TPP family genes also differed among different tissues (Figure 8C). *GhTPP8/19/20* were more highly expressed in the torus, and *GhTPP21/7/5/17* were highly expressed in roots, and *GhTPP7* was specifically expressed only in the roots. *GhTPP13/23/6/18/11/12/24/15* were most highly expressed in the anthers, which is consistent with previous reports that TPPs could regulate the development of floral organs by regulating the sucrose status of plants. Therefore, the expression of TPP family genes is induced by stress, but these genes may also participate in the growth and development processes of different organs of cotton.

### 2.9. Analysis of the Function of GhTPP22 in Cotton in Response to Stress

**Figure 9:**
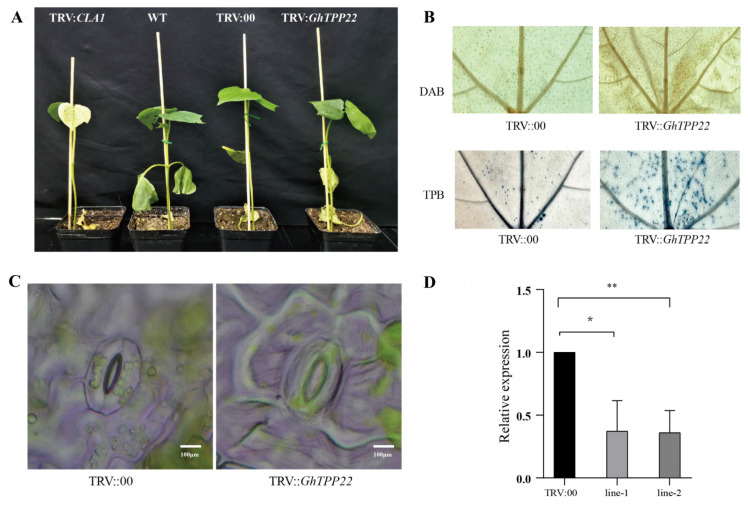
Functional study of *GhTPP22*. (**A**) Growth and development of TRV::*CLA1*, WT, TRV::00 and TRV::*GhTPP22* plants under drought stress; (**B**) DAB and trypan blue (represented in the figure as TPB) staining of TRV::00 and TRV::*GhTPP22* leaves; (**C**) The stomatal closure status of TRV::00 and TRV::*GhTPP22* under drought conditions; (**D**) qPCR verification results; the error bars show the standard deviations from three technical replicates, *and ** indicate the significant differences between the control samples (TRV::00) and the samples collected from TRV::GhTPP22 plants, as determined by Student’s *t*-test, at *p*-values ≤0.05 and≤0.01, respectively.

In this experiment, the effects of *GhTPP22* on plant development under drought stress were preliminarily studied due to its higher expression level. We constructed a TRV:*GhTPP22* virus-induced gene silencing (VIGS) vector and subjected the test plants to drought treatment. The results showed that after 15 days of drought treatment, TRV:00 and wild-type (WT) plants developed normally, whereas the leaves of *GhTPP22*-silenced plants were wilted (Figure 9A), which indicated that plants were more sensitive to drought stress after the silencing of *GhTPP22*. It was previously reported that genes tend to help plants resist drought stress through stomatal closure, thus, we assessed the stomatal closure of the tested plants. Most stomata of the TRV:*GhTPP22* plants were partially closed, and the degree of closure was greater in TRV:00 plants (Figure 9C). According to the qPCR assay results, the relative expression of *GhTPP22* in the TRV:*GhTPP22* plants was lower than that in the TRV:00 plants, which indicated that *GhTPP22* was silenced (Figure 9D).

Under drought stress, plants generate a large number of reactive oxygen species (ROS), which damage cells. In *Arabidopsis*, the ROS content was significantly higher in *AtTPPB* mutants under drought stress, whereas the ROS content was lower in plants overexpressing *AtTPPB*, which suggested that TPPs may protect the stability of cell membranes by interfering with the removal of ROS and thus participate in the drought resistance of plants. To verify whether the function of TPPs in cotton is the same as that of *AtTPPB*, the H_2_O_2_ content in the different lines was determined by the DAB staining of TRV:00 and TRV:*GhTPP22* leaves. The results showed that the TRV:*GhTPP22* plants exhibited larger brown areas and showed deeper staining, and trypan blue staining revealed more dead cells in the TRV:*GhTPP22* plants (Figure 9B), which indicated that the silencing of *GhTPP22* reduced the ability of plants to scavenge ROS and led to cell death.

## 3. Discussion

With the continuous development of sequencing technology, the genomes of *G. arboreum*, *G. raimondii*, *G. hirsutum*, and *G. barbadense* have been sequenced successively [20,21,24], and studies on gene families have shown gradual increases in detail and comprehensiveness. Here, we identified 17, 12, 24, and 26 TPP family genes in the *G. arboreum, G. raimondii*, *G. hirsutum*, and *G. barbadense* genomes, respectively, and carefully studied their structure and evolutionary selection, the cis-elements in their promoters and the physicochemical properties of their encoded proteins. *G. arboreum* and *G. raimondii* provided the At and Dt subgenomes for *G. hirsutum* and *G. barbadense,* respectively, via genome-wide replication events [20,25], which is the main method of gene family expansion, followed by tandem duplication, segmental duplication via retrotransposition and exon duplication and shuffling [26,27]. In this study, no tandem duplication was observed in the TPP gene family, but the genes may have undergone independent evolutionary processes in *G. hirsutum* and *G. barbadense* according to the collinearity analysis. Duplication events such as segmental replication have played critical roles in expanding the gene family members in plants [28,29]. In addition, mutation event coding sequences and regulatory regions can cause variations in the functions of gene family members [30,31].

Trehalose plays important roles in embryonic development, inflorescence formation, cellular morphogenesis and signal transduction. Differences in tissue expression are the result of selective gene expression. The expression of TPP genes, which encode the expression of the key enzyme in the last step of trehalose synthesis, directly affects the trehalose content. In this study, we analysed the expression patterns of TPP genes in different tissues of *G. hirsutum* and found that the expression of TPP genes exhibited significant differences among different tissues, with higher expression occurring mainly in the roots, torus and anthers, which is consistent with the results for *Arabidopsis* [32]. Moreover, TPP genes were highly expressed in the ovules from −3 to 3 DPA and 10 to 25 DPA in fibre, which suggested that these genes may also play a key role in fibre initiation and elongation. Trehalose exerts a specific effect on improving the resistance of plants to different stresses [33], but the amount of trehalose that accumulates in plants is not sufficient for it to act as a sole regulator in improving plant resistance; instead, trehalose is more likely to participate in signal transduction as a signalling molecule to regulate the expression of other downstream genes and improve stress resistance [16].

As the intermediate of the trehalose synthesis process, T6P is an important signal transduction mediator that participates in the regulation of plant growth and development, and the treatment of *Arabidopsis* with T6P can lead to stomatal closure and thus a response to drought stress. Plants with TPP gene silencing may accumulate T6P, which may lead to stomatal closure. Changes in the T6P content have an important impact on plant growth and development [34]. A mutant *TPS1* phenotype in *Arabidopsis* is reportedly lethal to embryos, and deletion of the TPS gene leads to a reduction in the T6P content in plants and thus results in abnormal growth. In addition, the accumulation of T6P in *Arabidopsis* under trehalose treatment can inhibit growth [35]. SnRK1s constitute a class of protein kinases that are widely present in eukaryotes, can sense the balance of energy and homeostasis in plants, and are involved in the stress response [36]. In plants, the accumulation of T6P can inhibit the activity of SnRK1 protein kinase [15,37,38], and in peas (*Pisum sativum*) specifically, it can inhibit the activity of SnRK1, and increase the concentration of T6P in embryos. The T6P/SnRK1 signalling pathway plays an extremely important role in signal regulation for plant sucrose metabolism [39]. For example, this pathway is involved in plant respiration, starch synthesis, starch and sucrose metabolism, and even ABA accumulation. Baena-González et al. [40] observed that overexpression of *AtKIN10* in *Arabidopsis* affected the inflorescence structure and also delayed the flowering period. By studying the growth of wheat at 10 DPA, Martinez-Barajas et al. [41] noted that the T6P/SnRK1 signalling pathway is involved in multiple growth and developmental stages of barley, including in the physiological regulation of grains, seeds coats and embryos, and can also regulate the “library” organs or cells of plants such as potato, sugarcane and cucumber [42,43,44]. In addition, a study found that *Arabidopsis* mutants lacking *TPS6* exhibited more inflorescence branches [45]. Therefore, decreased SnRK1 activity may indirectly affect the concentration of T6P. Under normal circumstances, after drought stress, TPP gene products consume T6P to synthesize trehalose, and SnRK1 activity increases, leading to increased resistance to drought stress. Thus, TPP genes may be involved in regulating the balance between T6P and SnRK1 in response to abiotic stress in plants. A recent report suggested that accumulated T6P may destroy actin organization, which would further impair the cell wall and influence fungal development and pathogenicity [46]. The above reports reveal the diversity of T6P functions. TPP genes can catalyse the decomposition of T6P to trehalose-6-phosphate and a phosphate group [32]; thus, we speculated that TPP genes may affect the normal growth of plants by regulating the concentration of T6P.

Stomatal closure is an important strategy used by plants to resist drought stress and is induced by a complex regulatory network. To date, the regulation of stomatal closure by trehalose and TPP genes has been reported only in *A. thaliana* [12,47]. The application of exogenous trehalose and ABA can lead to stomatal closure in plants with superimposed effects [47]. Water use efficiency is important for plants under drought stress [48]. Recent studies have shown that *AtTPPI* can reduce transpiration through stomatal closure and thereby improve water use efficiency [12]. In our experiment, *GhTPP22* silencing resulted in significantly increased stomatal opening compared with that in the control, and the TRV:*TPP22* plants were more sensitive to drought, which indicated that *GhTPP22* may have the same function in the resistance to drought stress in cotton.

Plants subjected to adverse stress generate ROS, resulting in oxidative stress and damage to cells. Studies have shown that TPP genes can respond to drought stress by inducing ROS scavenging. Some studies have also shown that ROS are involved in trehalose-induced stomatal closure [49], while another study found that *AtTPPE* stimulated metabolism by inducing the accumulation of ROS in roots [47]. Our findings support the former result. In the present study, the DAB staining results revealed substantial accumulation of ROS in the TRV:*TPP22* plants compared with the control plants. These results indicated that the ROS scavenging capacity decreased after gene silencing.

TPP family genes are key genes involved in trehalose synthesis, and their transcription and expression may be important for plant protection under adverse conditions. Members of the TPP gene family have been identified in *Arabidopsis* [11,12,50,51], rice [13,16,52], maize [10,18] and dicotyledonous short-stalked grass species [9], and among these, *Arabidopsis* and rice have been studied in greater detail. Overexpression of *OsTPP1* in rice can enhance tolerance to stress, and an analysis of overexpression lines showed that *OsTPP1* can trigger the expression of abiotic stress response-related genes, which suggested that *OsTPP1* stress-induced reprogramming may involve transcriptional regulatory pathways [16]. In this study, we used VIGS to silence *GhTPP22*, and the silenced plants were more sensitive to drought stress than the WT and TRV:00 plants. Moreover, qPCR assays revealed a reduction in *GhTPP22* expression. We believe that *GhTPP22* can respond to drought stress by regulating the excess ROS content in new leaves and the stomatal radius. However, whether the scavenging of ROS occurs due to osmoprotection caused by trehalose has not been definitively determined.

Trehalose can regulate the characteristics of stomatal movement and effectively induce stomatal closure, which is of great significance for enhancing plant stress resistance, reducing transpiration-related water loss and improving crop water use efficiency. The negative impacts of climate change on the ecological environment and social economy are becoming increasingly prominent. Countermeasures to reduce greenhouse gas emissions are urgently needed. A large number of studies, including this study, have shown that trehalose plays an important role in plant tolerance to abiotic stresses such as drought, high salinity and high temperature. However, because plant stress resistance is an extremely complex physiological process, it is controlled by multiple genes, and the effect of single-gene genetic transformation on improving plant stress resistance is limited. Sazzad et al. [53] proposed that the addition and application of promoters of TPS/TPP genes can solve the change in plant shape of TPS/TPP genes. It is one of the research hotspots to use the defence reactions of trehalose to breed varieties with drought and salt stresses. With the deepening understanding of the specific biological functions of trehalose, it is expected to play a more important role in crop stress resistance breeding.

## 4. Conclusions

In this study, 17, 12, 26, and 24 TPP family genes were identified in *G. arboreum*, *G. raimondii*, *G. barbadense* and *G. hirsutum*, respectively, and the chromosomal location, structure, and evolution of these TPP family genes in cotton as well as the physicochemical properties of their encoded proteins were analysed. The 79 TPP genes were divided into three groups by phylogenetic analysis. There was no tandem duplication in the TPP gene family, but different evolutionary directions were observed in *G. hirsutum* and *G. barbadense.* VIGS experiments showed that plants were more sensitive to drought stress after *GhTPP22* was silenced. Further research revealed that stomatal closure and scavenging of ROS decreased in silenced plants compared to the control. Overall, *GhTPP22* plays a key role in drought stress. Our experiment provides new ideas for improving drought resistance for cotton breeding in the future.

## 5. Materials and Methods

### 5.1. Identification and Physicochemical Properties of the TPP Gene Family in Cotton

The genome files, general feature format files (GFF3) and the protein sequences of *G. hirsutum* (CRI), *G. barbadense* (HAU), *G. arboreum* (CRI) and *G. raimondii* (JGI) were downloaded from the Cotton Functional Genomics Database (CottonFGD, https://cottonfgd.org/ (accessed on 13 April 2022)) [54]. Similarly, sequence information for 10 members of the *Arabidopsis* TPP family was downloaded from the *Arabidopsis* Information Resource (TAIR) database (https://www.arabidopsis.org/ (accessed on 13 April 2022)). The sequences of the TPP family genes in *Arabidopsis* were aligned with those in cotton, and the sequences of the corresponding TPP family genes in cotton were obtained. The obtained protein sequences were then queried in the Conserved Domain Database on the NCBI website (https://www.ncbi.nlm.nih.gov/Structure/bwrpsb/bwrpsb.cgi (accessed on 13 April 2022)) [55] to identify their conserved structural domains and further confirmed using the HMM search function in HMMER 3.0. [56]. Information on the isoelectric point and relative molecular mass of the proteins was obtained using an online tool (https://web.expasy.org/compute_pi/ (accessed on 13 April 2022)) [57], and MapChart software (https://www.wur.nl/en/show/Mapchart/ (accessed on 13 April 2022)) [58] was used to visualize the chromosomal locations of the TPP family genes. The CELLO program (http://cello.life.nctu.edu.tw/ (accessed on 13 April 2022)) [59] was used to predict subcellular localization.

### 5.2. Phylogenetic Analysis

The protein sequences of TPPgenes in cotton and *Arabidopsis* were used to construct the phylogenetic tree. The MUSCLE module of MEGA 7.0 [60] software was used to align the sequences of the TPP gene family members in cotton and identify the best base substitution model. The JTT + G model was selected, and the maximum likelihood (ML) method was used to construct the phylogenetic tree; the bootstrap value was set to 1000. The results were visualized by EvolView (https://evolgenius.info//evolview-v2/#login (accessed on 13 April 2022)).

### 5.3. Gene Structure and Conserved Motif Analysis

The MEME online tool (http://meme-suite.org/ (accessed on 13 April 2022)) [61] was used to analyse the conserved motifs of the protein sequences encoded by TPP gene family members. The maximum number of lookups was set to 10, and the other parameters were set to the default values.

### 5.4. Analysis of the Promoters of the TPP Genes in G. Hirsutum

TBtools [62] was used to extract the 2000-bp sequence upstream of the coding DNA sequence (CDS) of TPP family genes by genome sequence file and GFF3 file. The PlantCARE (http://bioinformatics.psb.ugent.be/webtools/plantcare/html/ (accessed on 13 April 2022)) [63] was used for the analysis, and the obtained results were organized and visualized using the Genome Structure Display Server (GSDS) (http://gsds.gao-lab.org/index.php (accessed on 13 April 2022)) [64] online tool.

### 5.5. Homology Analysis of the TPP Gene Family Members

The protein sequences of the whole genomes of all four cotton species were aligned using BLAST, and MCScanX [65] software was used for collinearity analysis of the whole genomes to obtain block information and gene pair information data, which were subsequently visualized by TBtools.

### 5.6. Expression Profile Analysis of the TPP Gene Family in Different Tissues of Cotton and under Stress Conditions

Expression levels (FPKMs) of *G. hirsutum* TPP gene family members in eight tissues (roots, stems, leaves, pistils, stamens, calyxes, petals, and receptacles), ovules, and fibres at different developmental stages and under four types of stress (cold, heat, drought and salt stress) were from PRJNA490626 [20] and downloaded from CottonFGD. The transcriptomic data were normalized via log_2_(1 + fragments per kilobase of transcript per million mapped reads (FPKM)).

### 5.7. Prediction of microRNAs (miRNAs)

The psRNATarget server (https://www.zhaolab.org/psRNATarget/ (accessed on 13 April 2022)) [66] was used to predict the target relationships between miRNA and TPP genes in *G. hirsutum.* The miRNA data were also provided by the website. Steps were as follows: The CDS sequence of TPP genes in *G. hirsutum* were submitted as target candidates, and then, the published miRBase of *G. hirsutum* was chosen. The default option was selected for other parameters. The results were visualized by Cytoscape [67].

### 5.8. Functional Validation of the GhTPP22 Gene in G. Hirsutum

Using *G. hirsutum* TM-1 as the material, the function of *GhTPP22* was preliminarily verified by VIGS. The 319-bp fragment of *GhTPP22* was amplified from the cDNA library to construct the TRV::*GhTPP22* vector and then transformed into *Agrobacterium tumefaciens* GV3101. TRV1, TRV:00, TRV:*CLA1* and TRV::*GhTPP22* were cultured overnight in LB medium supplemented with kanamycin and rifampicin (50 mg/L), and the cells were incubated in osmotic medium containing 10 mM MgCl_2_, 10 mM MES and 20 μM acetosyringone. After dark treatment for 3–6 h, *A. tumefaciens* medium containing TRV:00, TRV:*CLA1* and TRV::*GhTPP22* was mixed with TRV1 at 1:1 and injected into cotton cotyledons. The infiltrated plants were kept in darkness for one day at room temperature and then grown at 20 °C under a 16 h light/8 h dark cycle. The plants were kept without water to simulate drought conditions. After 15 days of VIGS treatment, the leaves of the cotton treatment group and control group were carefully removed and placed in l mg/mL 3,3’-diaminobenzidine (DAB) staining solution (pH = 3.8) overnight. The staining solution was then removed, and the leaves were decolorized with 96% ethanol for 12 h. The leaves were suspended in water, and a stereomicroscope was used to observe and photograph the leaves. Three sets of samples from different plants (15 days after injection) were collected as three biological replicates. RNA was extracted from each group of samples and reverse-transcribed to cDNA using Full-Style Gold EasyScript^®^ One-Step gDNA Removal and cDNA Synthesis SuperMix. The same design was repeated three times for quantitative analysis. The qPCR tests were performed on QuantStudio 6 Flex thermocyclers (Applied Biosystems, Foster City, CA, USA) with a total volume of 10 μL, the protocol was as follows: (1) 95 °C for 5 min; (2) 95 °C for 30 s, 58 °C for 30 s, and 72 °C for 45 s for 40 cycles; and (3) 72 °C for 10 min. *GhHistone3* was used as the endogenous standard control. The 2^−ΔΔCT^ method [68] was used to calculate the relative expression level of TPP genes. The data were analysed by a *t*-text using GraphPad Prism. All the primers used in this experiment are listed in Table S3.

## Figures and Tables

**Table 1:** Basic information of TPP family genes in cotton.

Gene ID	Gene Name	Amino Acid Length	PI	MW (Da)	Subcellular Location	Location
*Ga01G0805*	*GaTPP1*	197	8.79	22,151.11	chloroplast	Ga01:12056674−12058006(−)
*Ga01G2073*	*GaTPP2*	415	5.53	46,497.55	chloroplast	Ga01:98691188−98693520(+)
*Ga01G2696*	*GaTPP3*	387	8.79	43,611.07	cytoplasm, mitochondrion	Ga01:111288363−111290680(+)
*Ga02G0355*	*GaTPP4*	365	9.01	41,110.26	endoplasmic reticulum	Ga02:4283490−4285630(+)
*Ga02G1338*	*GaTPP5*	349	9.37	39,003.96	mitochondrion	Ga02:91984769−91987027(+)
*Ga02G1426*	*GaTPP6*	307	7.13	34,427.13	chloroplast	Ga02:93890804−93892792(+)
*Ga04G1370*	*GaTPP7*	174	9.15	19,797.69	cell membrane	Ga04:82923593−82924930(−)
*Ga05G2426*	*GaTPP8*	369	9.31	41,176.46	chloroplast	Ga05:22943084−22945173(−)
*Ga06G0361*	*GaTPP9*	364	9.03	40,884.08	peroxisome	Ga06:4054349−4056277(−)
*Ga08G2961*	*GaTPP10*	248	9.3	28,235.55	chloroplast	Ga08:129160428−129168443(−)
*Ga09G2059*	*GaTPP11*	327	6.13	36,864.31	chloroplast	Ga09:78465375−78467180(−)
*Ga10G0429*	*GaTPP12*	181	9.05	20,501.79	chloroplast	Ga10:6841862−6843187(+)
*Ga11G1620*	*GaTPP13*	370	9.03	41,573.91	nucleus	Ga11:66146228−66148090(−)
*Ga12G0284*	*GaTPP14*	340	9.01	38,454.32	endoplasmic reticulum	Ga12:2329765−2331423(−)
*Ga12G0607*	*GaTPP15*	388	8.47	43,856.03	mitochondrion	Ga12:5363443−5365648(+)
*Ga12G0815*	*GaTPP16*	383	7.69	42,710.69	chloroplast	Ga12:7602925−7605180(−)
*Ga12G1996*	*GaTPP17*	365	9.17	41,099.14	chloroplast	Ga12:36111089−36113512(+)
*Gorai.002G219000*	*GrTPP1*	354	9.57	39,276.39	mitochondrion	Gr02:57218239−57220778(+)
*Gorai.003G038300*	*GrTPP2*	373	9.11	41,994.42	endoplasmic reticulum	Gr03:4115747−4118444(+)
*Gorai.003G113900*	*GrTPP3*	419	5.62	46,886.94	chloroplast	Gr03:34671718−34674357(+)
*Gorai.003G169300*	*GrTPP4*	387	8.79	43,600.99	cytoplasm, mitochondrion	Gr03:43992274−43996267(−)
*Gorai.006G200800*	*GrTPP5*	327	6.35	37,063.49	chloroplast	Gr06:45780176−45782343(−)
*Gorai.007G240600*	*GrTPP6*	370	9.23	41,706.1	nucleus	Gr07:33289543−33291870(−)
*Gorai.008G104600*	*GrTPP7*	377	9.13	42,485.96	chloroplast	Gr08:32381215−32384204(−)
*Gorai.008G216700*	*GrTPP8*	422	8.4	47,239.17	chloroplast	Gr08:50362362−50368208(+)
*Gorai.008G236800*	*GrTPP9*	388	8.47	43,922.1	mitochondrion	Gr08:52268635−52273612(−)
*Gorai.008G270100*	*GrTPP10*	340	8.87	38,339.14	endoplasmic reticulum	Gr08:54895162−54901806(+)
*Gorai.009G238600*	*GrTPP11*	369	9.31	41,190.49	chloroplast	Gr09:18961184−18963904(−)
*Gorai.010G042600*	*GrTPP12*	364	9.24	40,853.11	peroxisome	Gr10:4054502−4056819(−)
*Gbar_A01G006620*	*GbTPP1*	313	8.54	35,142.68	chloroplast	A01:10624088−10627713(−)
*Gbar_A01G018380*	*GbTPP2*	365	9.55	41,026.49	mitochondrion	A01:108393899−108396649(+)
*Gbar_A01G019190*	*GbTPP3*	247	8.45	27,951	vacuole	A01:110286108−110293672(+)
*Gbar_A02G015580*	*GbTPP4*	365	9.05	41,062.2	endoplasmic reticulum	A02:95555407−95558198(−)
*Gbar_A03G001550*	*GbTPP5*	387	8.79	43,611.07	cytoplasm, mitochondrion	A03:1858338−1862358(+)
*Gbar_A03G006910*	*GbTPP6*	377	5.39	42,113.75	peroxisome	A03:13211987−13217500(−)
*Gbar_A05G022530*	*GbTPP7*	369	9.31	41,220.52	chloroplast	A05:22046615−22049049(−)
*Gbar_A06G003690*	*GbTPP8*	364	9.03	40,884.08	peroxisome	A06:4817982−4820242(−)
*Gbar_A06G003820*	*GbTPP9*	239	8.56	26,686.69	mitochondrion	A06:5644700−5662178(+)
*Gbar_A08G027440*	*GbTPP10*	259	6.05	28,424.14	nucleus	A08:119673859−119677052(−)
*Gbar_A09G019400*	*GbTPP11*	327	6.13	36,909.37	chloroplast	A09:71628362−71630477(−)
*Gbar_A11G020620*	*GbTPP12*	370	9.03	41,588.89	nucleus	A11:42027092−42029383(−)
*Gbar_A12G021250*	*GbTPP13*	383	7.69	42,710.69	chloroplast	A12:94907154−94913205(+)
*Gbar_A12G023290*	*GbTPP14*	388	8.47	43,870.05	mitochondrion	A12:97098300−97103172(−)
*Gbar_A12G026200*	*GbTPP15*	340	8.92	38,427.25	endoplasmic reticulum	A12:99713201−99720168(+)
*Gbar_D01G019570*	*GbTPP16*	354	9.57	39,306.42	mitochondrion	D01:57326568−57329308(+)
*Gbar_D03G003620*	*GbTPP17*	373	9.12	41,978.42	endoplasmic reticulum	D03:4085281−4087934(+)
*Gbar_D03G011280*	*GbTPP18*	384	5.77	42,710.49	peroxisome	D03:39684077−39689078(+)
*Gbar_D03G016980*	*GbTPP19*	387	8.96	43,682.05	cytoplasm, mitochondrion	D03:49371797−49375803(−)
*Gbar_D05G023190*	*GbTPP20*	369	9.31	41,156.47	chloroplast	D05:20587648−20591044(−)
*Gbar_D06G003870*	*GbTPP21*	364	9.23	40,886.18	chloroplast	D06:4415421−4417669(−)
*Gbar_D11G023430*	*GbTPP22*	370	9.1	41,707.04	nucleus	D11:35062160−35064485(−)
*Gbar_D12G010160*	*GbTPP23*	360	9.25	40,613.84	mitochondrion	D12:33516324−33519721(−)
*Gbar_D12G021190*	*GbTPP24*	383	8.52	42,648.71	chloroplast	D12:51949877−51955716(+)
*Gbar_D12G022910*	*GbTPP25*	388	8.47	43,922.1	mitochondrion	D12:53800044−53805108(−)
*Gbar_D12G026150*	*GbTPP26*	340	8.74	38,360.16	endoplasmic reticulum	D12:56766186−56773133(+)
*Gh_A01G209200*	*GhTPP1*	365	9.55	41,026.49	mitochondrion	A01:108650087−108652331(+)
*Gh_A02G174500*	*GhTPP2*	365	9.05	41,062.2	endoplasmic reticulum	A02:101053973−101056656(−)
*Gh_A03G016000*	*GhTPP3*	387	8.99	43,610.13	cytoplasm, mitochondrion	A03:1987850−1990163(+)
*Gh_A03G074500*	*GhTPP4*	415	5.65	46,558.73	chloroplast	A03:14292959−14295295(−)
*Gh_A05G212200*	*GhTPP5*	369	9.31	41,190.49	chloroplast	A05:22966211−22968675(−)
*Gh_A06G040600*	*GhTPP6*	364	9.03	40,884.08	peroxisome	A06:4961458−4963382(−)
*Gh_A06G042800*	*GhTPP7*	134	5.87	14,881.02	cytoplasm	A06:5763598−5765589(+)
*Gh_A09G201500*	*GhTPP8*	327	6.13	36,864.31	chloroplast	A09:77041091−77042896(−)
*Gh_A11G215100*	*GhTPP9*	339	9.05	38,310.18	endoplasmic reticulum, mitochondrion, plastid	A11:44824072−44825936(−)
*Gh_A12G223300*	*GhTPP10*	383	7.69	42,710.69	chloroplast	A12:98041663−98047469(+)
*Gh_A12G243500*	*GhTPP11*	388	8.47	43,870.05	mitochondrion	A12:100217966−100222660(−)
*Gh_A12G275900*	*GhTPP12*	340	8.92	38,427.25	endoplasmic reticulum	A12:103292432−103294090(+)
*Gh_D01G206700*	*GhTPP13*	354	9.57	39,306.42	mitochondrion	D01:58776685−58778828(+)
*Gh_D03G037900*	*GhTPP14*	365	9.12	41,103.3	endoplasmic reticulum	D03:4191881−4194554(+)
*Gh_D03G122800*	*GhTPP15*	422	5.79	47,156.41	chloroplast	D03:41577803−41580170(+)
*Gh_D03G179600*	*GhTPP16*	387	8.65	43,629.99	cytoplasm, mitochondrion	D03:51536913−51539437(−)
*Gh_D05G229100*	*GhTPP17*	369	9.19	41,157.41	chloroplast	D05:20569568−20571657(−)
*Gh_D06G040500*	*GhTPP18*	364	9.23	40,886.18	chloroplast	D06:4645870−4648178(−)
*Gh_D09G194700*	*GhTPP19*	327	6.14	37,129.59	chloroplast	D09:46877954−46879754(−)
*Gh_D11G237500*	*GhTPP20*	370	9.11	41,657.97	nucleus	D11:35983647−35985917(−)
*Gh_D12G107500*	*GhTPP21*	377	9.23	42,452.94	chloroplast	D12:36644512−36646944(−)
*Gh_D12G217800*	*GhTPP22*	383	8.52	42,648.71	chloroplast	D12:55343343−55349138(+)
*Gh_D12G238200*	*GhTPP23*	388	8.47	43,956.12	mitochondrion	D12:57317788−57319992(−)
*Gh_D12G270400*	*GhTPP24*	340	8.74	38,360.16	endoplasmic reticulum	D12:60342947−60344621(+)

## Data Availability

The genome of four cotton species and RNA-seq data were downloaded from the CottonFGD website (https://cottonfgd.org/ (accessed on 13 April 2022)).

## Supplementary Materials

The following supporting information can be downloaded at: https://www.mdpi.com/article/10.3390/plants11081079/s1, Table S1: The information of motifs; Table S2: The prediction details of cis-elements; Table S3: The primers in this experiment.
